# Therapeutic evaluation of HIV transduction basic domain-conjugated superoxide dismutase solution on suppressive effects of the formation of peroxynitrite and expression of COX-2 in murine skin

**DOI:** 10.1186/s12929-016-0226-7

**Published:** 2016-01-20

**Authors:** Tsang-Pai Liu, Yi-Ping Chen, Chih-Ming Chou, Ting-Ting Chiu, Chien-Tsu Chen

**Affiliations:** Mackay Junior College of Medicine, Nursing and Management, Taipei, Taiwan; Department of Surgery, Mackay Memorial Hospital, Taipei, Taiwan; Graduate Institute of Nanomedicine and Medical Engineering, College of Biomedical Engineering, Taipei Medical University, Taipei, Taiwan; Department of Biochemistry and Molecular Cell Biology, College of Medicine, Taipei Medical University, Taipei, Taiwan

**Keywords:** Denatured Tat-SOD, TPA, COX-2, SNP, Skin disease, Protein therapy

## Abstract

**Background:**

Homeostasis of reactive oxygen species (ROS) in the skin is regulated by antioxidant defenses. The inflammatory states of skin diseases which range from acute rashes to chronic conditions are related to the level of ROS. The involvement of superoxide dismutase (SOD) in restoring the antioxidant capacity can then neutralize the inflammatory response.

**Results:**

We found that denatured Tat-SOD formulated in an aqueous medium could be delivered into mouse skin and the penetration signals of Tat-SOD were detected in the epidermis and dermis. According to immunohistochemical staining, Tat-SOD successfully suppressed inflammation induced by 12-O-tetradecanoylphorbol-13-acetate (TPA), the expression of sodium nitroferricyanide (SNP)-induced cyclooxygenase-2 (COX-2), and the production of nitrotyrosine proteins. In nerve growth factor (NGF) induced differentiated PC12 pheochromocytoma cells, we demonstrated that the denatured Tat-SOD regained its antioxidant activity and effectively protected PC12 cells from DNA fragmentation induced by paraquat. Using a luciferase reporter assay, the data was shown Tat-SOD protected PC12 cells from ROS damage, through suppression of COX-2 or nuclear factor-κB (NF-κB) activity occurred at the transcriptional level.

**Conclusion:**

We showed that Tat-SOD inhibited SNP-induced COX-2 expression similarly to celecoxib and prevented the formation of peroxynitrite as 2-phenyl-4,4,5,5-tetramethylimidazoline-1-oxyl-3-oxide. The results suggest that denatured Tat-SOD solution may perform potential protein therapy for patients suffering from disorders related to ROS.

## Background

In physiological surroundings, the skin is constantly exposed to free radicals generated from external and internal circumstances. The buildup of reactive oxygen species (ROS) in the skin is determined by numerous antioxidant defense reactions, including both enzymatic and non-enzymatic mechanisms that either prevent the formation of ROS or scavenge those ROS generated. However, inflammatory conditions which overwhelm the antioxidant defense system can cause abnormal ROS levels that raise oxidative stress to pathological levels, resulting in deterioration of cells into diseased states, ranging from acute rashes with itching and redness, chronic syndromes like dermatitis (eczema) to psoriasis and even burn trauma, by way of oxidative modifications that alter biomolecular functions and structure and dysregulate cell signaling that results in aberrant cytokine release [1]. For example, acute sunburn induced by UV-radiation opens up vulnerable skin to attack by excessive ROS due to depletion of antioxidant defenses [2–4]. Psoriasis, a chronic inflammatory skin disease, was also suggested to involve unregulated ROS levels, and its etiology is related to dysregulation of inflammatory pathways, like nuclear factor-κB (NF-κB), mitogen-activated protein kinase (MAPK) and JAK-STAT [5–8]. Burn injuries, a posttraumatic inflammatory condition, are thought to involve oxygen restoration which promotes superoxide production to exacerbate ischemic tissue injuries by increasing lipid peroxidation products, conjugated dienes, or malondialdehyde. Meanwhile, burns induce the upregulation of inducible nitric oxide (NO) synthase (iNOS), thus increasing the production of NO for peripheral vasodilatation, activation of the transcription factor NF-κB, and responses by numerous cytokines. NO highly reacts with superoxide anions to produce peroxynitrite, a risky mediator of cell and tissue injury [9–11]. Reducing oxidative stress by producing homeostasis through regulating ROS levels can then neutralize the inflammatory response [12–14].

Since the skin consists principally of a layer of vascular connective tissue (namely, the corium or cutis vera) and an external covering of epithelium tissue (namely, the epidermis or cuticle) with the peripheral endings of many sensory nerves, the sudoriferous, sebaceous glands and the hair follicles, it has limited excretory and absorbing powers. Some injuries that may affect all layers of the skin to cause significant cell damages by the subsequent inflammatory response. Competent therapeutic approaches to restore the antioxidant capacity in the skin can be achieved by preventing the formation of ROS, strengthening endogenous antioxidant enzymatic activity, and scavenging ROS using pharmacological or dietary supplements. Superoxide dismutase (SOD, EC 1.15.1.1) catalyzes the dismutation of superoxide and is considered a therapeutic protein for treating human inflammatory diseases. In several countries, SOD (orgotein) is locally injected for relief of sports injuries, osteoarthritis, and several inflammatory symptoms [15, 16]. Among available formulations of SOD, a liposomal-encapsulated native SOD injection was effective in treating systemic inflammatory diseases and skin ulcer lesions, especially those from wounds and burns [9, 17, 18]. Topical application of native Cu,Zn-SOD was also effective against skin lesions, antipruritic activity, and burns with skin transplantation [19, 20]. However, native SOD formulated by dissolving it in a vehicle of petrolatum or water did not retain its activity for long periods [9, 21, 22].

Several in vivo studies by intraperitoneal injection or oral administration of Tat-SOD indicated that the Tat-SOD has been observed the distribution in rats and mice, including liver, kidney, heart and brain. Topical application on mice ears of Tat-SOD has been found to suppress TPA-induced expression of proinflammatory cytokines and enzymes regulated by reducing the activation of NF-κB and MAPK [23]. Increase of intracellular SOD activity resulting in decreasing the ROS induced damage was also reported in mammalian cells [24–27]. We previously constructed a human Cu,Zn-SOD (SOD1) fused with a nine-amino-acid (RKKRRQRRR) transactivator of transcription domain (Tat 49-57) of human immunodeficiency virus (HIV) type 1 in a bacterial vector to produce a Tat-SOD recombinant protein. The exogenous Tat-SOD, a novel modified form of SOD, exhibited direct cellular transduction into undifferentiated PC12 pheochromocytoma cells and a cellular protective function against oxidative stress from paraquat-induced cell death [28]. In the other study, we found that Tat-SOD is conjugated to mesoporous silica nanoparticles (MSNs) to form MSN-Tat-SOD. The data showed that MSN-Tat-SOD effectively reduces cell apoptosis in HeLa cells through an endosomal escape action [29].

The aim of the present study was to develop an effective therapy using the denatured Tat-SOD solution. We first demonstrated that topical application of the denatured Tat-SOD protein delivered in vivo into murine skin and the data showed that Tat-SOD exhibited multiple effects of suppressing TPA-induced inflammation, SNP-induced cyclooxygenase-2 (COX-2), and nitrotyrosine production. Then, we also demonstrated Tat-SOD protein in vitro in differentiated PC12 cells suggested increasing intracellular SOD activity to protect neuronal cells from the ROS induced damage. The suppression of COX-2 or NF-κB expression by Tat-SOD was attributable to inhibition at the transcriptional level. The results suggest that denatured Tat-SOD solution may perform potential protein therapy for patients suffering from disorders related to ROS.

## Methods

### Chemicals

Isopropyl-L-D-thiogalactopyranoside (IPTG) and a luciferase assay kit were purchased from Promega (Madison, WI). Sodium nitroferricyanide (SNP), 12-O-tetradecanoylphorbol-13-acetate (TPA), indomethacin (Indo), and 2-phenyl-4,4,5,5-tetramethylimidazoline-1-oxyl-3-oxide (PTIO) were purchased from Sigma (St. Louis, MO). Ni^2+^-nitrilotriacetic acid (NTA) Sepharose superflow was purchased from Qiagen (Valencia, CA). Lastly, celecoxib (CELEBREX™) was from Phamacia (Northumberland, UK).

### Preparation of denatured recombinant Tat-SOD fusion proteins

Expression and purification of recombinant Tat-SOD fusion proteins were according to previous study [28, 30]. The recombinant Tat-SOD fusion proteins were purified under denaturing condition. To denature Tat-SOD fusion proteins, harvested cells were disrupted by sonication in a phosphate buffer (300 mM NaCl, and 50 mM NaH_2_PO_4_, pH 8) containing 8 M urea. After centrifugation, supernatants containing Tat-SOD fusion proteins were collected and purified by Ni^2+^-NTA Sepharose metal affinity resins according to the manufacturer’s instructions (Qiagen, Valencia, CA, USA). The purified denature-form proteins were concentrated and the salts were removed using Amicon® centrion (10 kDa MWCO,Millipore, Bedford, MA, USA). The concentrated and desalted proteins were estimated by the Bradford procedure using bovine serum albumin as a standard. Finally, the purified denatured proteins were dissolved in PBS containing 20 % glycerol and then aliquoted and stored at -80 °C.

### Preparation of quantum dots conjugated with Tat-SOD

CdSe/ZnS quantum dots (560 nm; Nano Ocean Tech, Springdale, AR) were dissolved in chloroform and mixed with an equal volume of hydrating solution (distilled water: ethanol = 1: 1, 10 mg/mL sodium thioglycolate, and 2 μL ammonia). After the mixture was stirred for 1 h, the water layer was washed with the same volume of acetone and centrifuged at 5000 rpm. The pellet was dried and suspended in phosphate-buffered saline (PBS). The quantity of quantum dots was determined by a spectrophotometric method. The calculation followed Beer’s rule with a coefficient factor of 1.7 × 10^5^. Equal moles of quantum dots and denatured Tat-SOD were mixed and incubated for 1 h before cell transduction.

### Cell lines and culture conditions

PC12 cells, from a rat pheochromocytoma cell line, were obtained from American Type Culture Collection (Manassas, NJ) and cultured as recommended at 37 °C in Dulbecco’s modified Eagle medium (DMEM; GIBCO, Life Technologies Inc.) supplemented with 5 % heat-inactivated fetal bovine serum (FBS; HyClone, Logan, UT), 10 % horse serum, penicillin G (100 U/mL), streptomycin (100 μg/mL), and L-glutamine (2 mM) in a humidified atmosphere of 5 % CO_2_. Other reagents were purchased from HyClone.

### Western blot analysis

PC12 cell lysates were separated on 10 % sodium dodecylsulfate polyacrylamide gel electrophoresis (SDS-PAGE), and proteins were then electrophoretically transferred to a polyvinylidene difluoride (PVDF) membrane. The membrane was incubated 1 h at room temperature in blocking buffer (1× Tris-buffered saline-0.1 % Tween 20, and 5 % w/v nonfat dry milk), and then incubated with a polyclonal anti-human Cu,Zn-SOD antibody for 16 h at 4 °C. The membrane was extensively washed and incubated with a horseradish peroxidase-conjugated goat anti-rabbit immunoglobulin G antibody (diluted 1:3000; Jackson, West Grove, PA) for 1 h at room temperature. Immunoreactive bands were visualized with an enhanced chemiluminescence substrate kit (NEN, Boston, MA) according to the manufacturer’s protocol.

### Immunocytochemical staining for Tat-SOD expression

PC12 cells were fixed with 4 % paraformaldehyde, and permeabilized with 0.1 % Triton X-100 in PBS. After being washed with PBS, cells were blocked in blocking buffer for 1 h and then incubated with a polyclonal anti-human Cu,Zn-SOD antibody for 16 h at 4 °C. Cells were extensively washed and visualized with an anti-rabbit Cy3-labeled secondary antibody at a 1: 1000 dilution for 2 h, and nuclei were counterstained with DAPI (a DNA marker) for 5 min. Cells were analyzed with an Olympus IX70-FLA inverted fluorescence microscope. Images were taken using the SPOT system (Diagnostic Instruments, Sterling Heights, MI).

### DNA fragmentation analysis

Cells were lysed for 3 h at 50 °C in lysis buffer (50 mM Tris–HCl, 10 mM EDTA, 0.5 % N-lauryl-sarcosine, and 0.5 mg/mL proteinase K), and then supplemented with an RNase solution in lysis buffer for 3 h at 50 °C. DNA was precipitated with phenol/chloroform (1: 1). DNA samples were then separated on a 1 % agarose gel, and visualized under UV illumination with staining.

### Determination of SOD activity

SOD activities of cell lysates were measured according to a method of Marklund [31]. The assay mixture, containing 500 μL buffer (50 mM Tris-cacodylic acid buffer at pH 8.4, 1 mM diethylenetriamine pentaacetic acid, and 1 mM phosphate buffer at pH 7.0), 50 μL of sample, 50 μL of pyrogallol, and 400 μL of water, was measured at an optical density of 420 nm. The increase in absorbance was measured for 3 min. SOD-specific activity was expressed as units per milligram (U/mg) of protein.

### Inductively coupled plasma atomic emission spectroscopy (ICP-AES)

The plasma technology used with mass spectrometry (ICP-MS) for simultaneous trace element determination (Zn, Cu, Hg, Cd, and As) in recombinant proteins was carried out as the most suitable method [32]. The water was Milli-Q grade (>18 MΩ). Equipment parameters, such as the torch position, voltages of the ionic lenses, and also the voltage of the detector and nebulization systems, were optimized and followed the manual instructions: power of 1.5 kW; plasma flow of 15 L/min; and auxiliary flow of 1.5 L/min. For calibration, a copper standard (1000 ppm, Merck, Darmstadt, Germany) was diluted with 0.15 % HNO_3_ to 0 ~ 50 ppb for use.

### Circular dichroism (CD) measurements

Proteins for the CD analysis were dissolved in PBS to a concentration of 2.5 μg/μL. CD spectra were measured with a Jasco J-715 spectropolarimeter, and CONTIN software was used to analyze the data. The mean residue ellipticity was estimated from the mean residue weight, which was calculated from the primary structure [30].

### Luciferase assay for COX-2 and NF-κB promoter activities

PC12 cells were transfected with a COX-2 or NF-κB luciferase reporter plasmid using Lipofectamine 2000 (Life Technologies Inc.) according to the manufacturer’s recommendations. At 24 h after transfection, cells were treated with Tat-SOD (0, 0.5, 1.0, 1.5, 2.0 μM), indomethacin (20 μg/mL), or PTIO (50 mM) for 2 h before the addition of TPA (50 ng/mL) or SNP (50 mM) for 4 h. Both firefly luciferase (FL) and *Renilla* luciferase (RL) activities were measured using a dual luciferase reporter assay kit (Promega Co.). The FL activity was normalized to the RL activity.

### Animal treatments

All experimental procedures were designed to minimize the total number of animals used and animal suffering during sacrifice of animal through cervical dislocation. All rules and regulations were followed during experimentation on animals and were approved by the local Animal Care Committee. All animal procedures were approved by the Institutional Animal Care and Use Committee (IACUC) of Taipei Medical University. (protocol #LAC-2013-0293). Six or seven-week-old female ICR mice were used as the experimental animals and purchased from the Laboratory Animal Center (National Taiwan University, Taipei, Taiwan). Animals were maintained in a room temperature and humidity-controlled facility with a 12-h light/dark cycle. A day before the experiments, the dorsal side skin was shaved using electric clippers. All samples in 0.05 mM CuCl_2_ and ZnCl_2_ were diluted with acetone and applied using an adjustable pipette (1 cm in diameter). The indicated doses of vehicle (acetone, 200 μL/site), Tat-SOD, celecoxib, or PTIO were topically administered to the shaven back of a mouse for 2 h, and then it was further treated with TPA (10 nmol/200 μL/site) [33] or SNP (20 μmol/200 μL/site) for 4 h. Finally, mice were sacrificed by cervical dislocation, and skin samples (1 cm in diameter) were obtained from the central dorsum of the mice for further experiments.

### Immunohistochemical (IHC) staining of COX-2 and nitrotyrosine protein in mouse skin

Sections (4 μm) of formalin-fixed, paraffin-embedded tissue were collected onto silcanized glass slides and deparaffinized. For antigen retrieval, sections were boiled in 10 mM citrate buffer (pH 6.0) for 10 min. Each section was treated with 3 % hydrogen peroxide in methanol for 15 min and incubated with 2 % normal goat serum for 30 min. Sections were incubated with a primary polyclonal anti-COX-2 (Cayman, Ann Arbor, MI) or polyclonal anti-nitrotyrosine (Upstate, Lake Placid, NY) antibody. The section was developed using DAB (HPR EnVisionTM system, Dako, Glostrup, Denmark) and counterstained with Mayer’s hematoxylin.

### Histological analysis of mouse skin

Skin samples were fixed with 4 % paraformaldehyde and then histologically stained with hematoxylin and eosin (H&E). Images were captured using an Olympus DP-70 camera on a Nikon ECLIPSE E800 microscope (at an original magnification of × 200).

### Statistical analysis

Experiments were repeated at least 3 times with similar results. Statistical significance was determined using Student’s *t*-test as indicated in the legend. *P*-values are reported to 4 decimal points, and results are expressed as mean ± standard error of the mean (sem). (*P < 0.05, **P < 0.01, ***P < 0.001, ****P < 0.0001).

## Results

### Preparation of denatured recombinant Tat-SOD

**Fig. 1:**
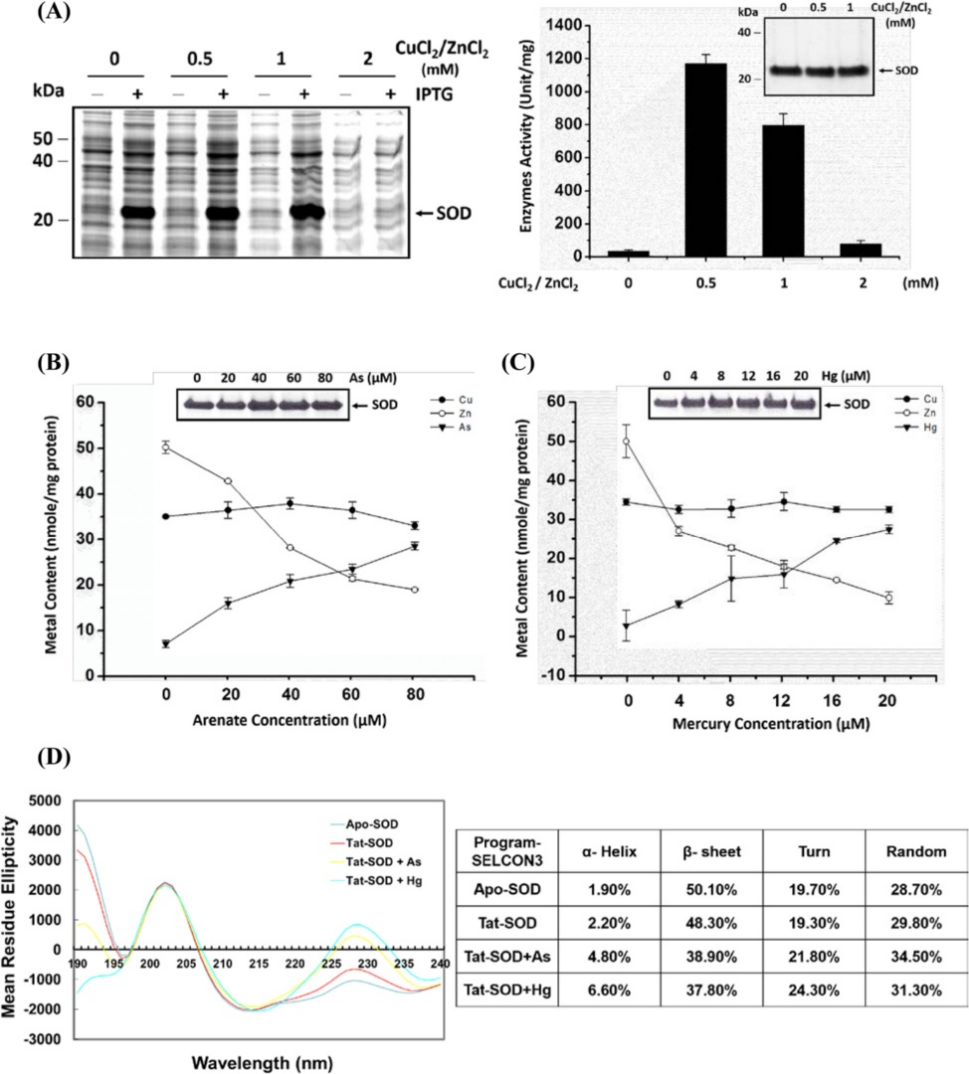
Measurement of heavy metals in Tat-SOD using an ICP-AES and circular dichroism analysis of Tat-SOD. **a** Overexpression of the antioxidant Cu,Zn-SOD gene in an *E. coli* expression system. Tat-SOD was expressed in a metal-dependent manner. **b** Identification the contents of copper, zinc, and arsenate in *E. coli* expressed Tat-SOD by incubating with 0, 20, 40, 60 or 80 μM of arsenic ions as indicated were analyzed by ICP-AES. **c** Identification the contents of copper, zinc, and mercury in *E. coli* expressed Tat-SOD by incubating with 0, 4, 8, 12, 16 or 20 μM of mercury ions as indicated were analyzed by ICP-AES. **d** Effects of arsenate and mercury on the secondary conformation of the SOD protein using a circular dichroism analysis (with a Jasco J-715 spectrometer)

The SOD protein manufactured for pharmaceutical approaches and clinical use was recently obtained through biotechnological sources. We have developed an efficient system to express and purify the new modified SOD protein, Tat-SOD. The constructed pQE-Tat-SOD expression plasmid containing sequences encoding SOD1, the Tat 49–57 domain, and the His-tagged peptide was transformed into *E. coli* JM109 and overexpressed by adding IPTG at a final concentration of 1 mM for 1 h [28]. The harvested protein with a specific activity of 1172 ± 229 SOD units/mg was purified by nickel Sepharose and assayed according to a method by Marklund [31]. Data in Fig. 1a show that Tat-SOD expression was metal-ion dependent. The concentration of metal ions for optimal SOD activity was about 0.5–1 mM of CuCl_2_ and ZnCl_2_. When CuCl_2_ and ZnCl_2_ exceeded 1 mM, protein expression decreased due to cytotoxic tolerance. The production of Tat-SOD was found to severely interfere with cadmium ions due to substitution of zinc ions necessary for catalytic activity inside the SOD molecule [30]. We herein investigated the heavy metals mercury and arsenate to the expression of SOD to evaluate how the metals affected its activity. In Fig. 1b and c, both mercury and arsenate ions, in the presence of CuCl_2_ and ZnCl_2_, displaced the zinc content of SOD molecules similar to that by cadmium ions and changed the SOD conformation according to circular dichroism data (Fig. 1d). Concentrations of 40 μM arsenate (III), 8 μM mercury (II) ions, and 150 nM cadmium (II) ions significantly inhibited SOD activity by half (IC_50_). This suggests that during the preparation and formulation of native SOD, some metals must be avoided. Therefore, using a denatured form of SOD is a strategic choice for therapy which takes advantage of reduced costs to keep it native. Hence, Tat-SOD purified with nickel beads was quantitated by the Bradford test and treated with 8 M urea to denature it. We formulated the denatured Tat-SOD solution in an isotonic aqueous medium for topical use.

### Tat-SOD attenuated SNP- and TPA-induced stress via cellular regulation of COX-2 and NF-κB in murine skin

**Fig. 2:**
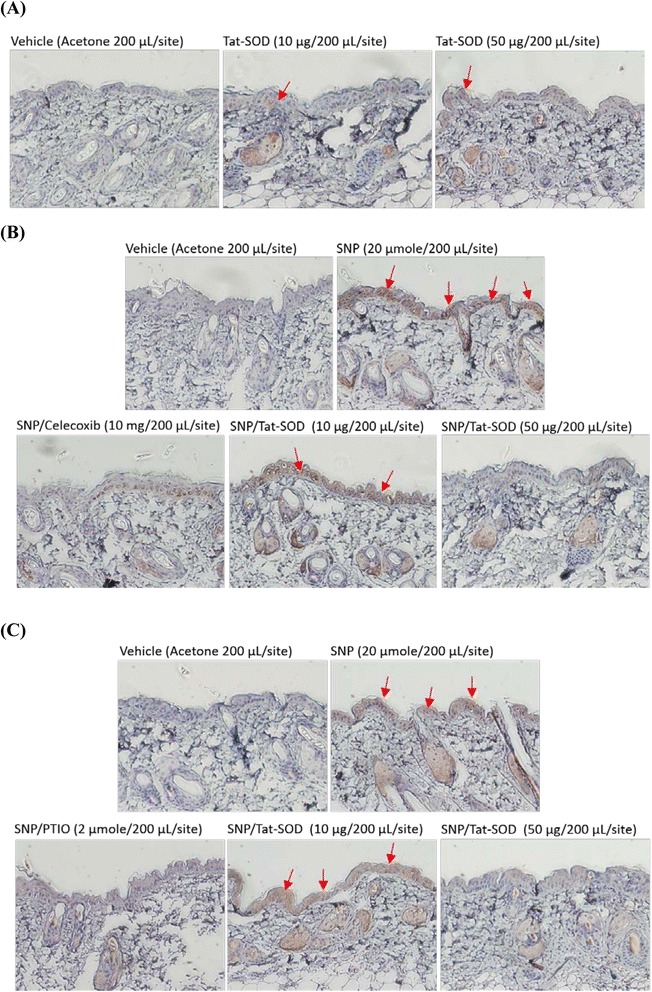
Transduction of the denatured Tat-SOD protein into mouse skin and IHC staining of SNP-induced cyclooxygenase-2 (COX-2) and nitrotyrosine protein expression. Dorsal skins of female ICR mice were treated topically with acetone (as the vehicle control) or indicated concentrations of Tat-SOD, celecoxib, or PTIO for 2 h before SNP (20 μmol/200 μL/site) treatment for 4 h. All samples were applied to the backs of animals in 200 μL acetone using an adjustable pipette (1 cm in diameter) and the effect of Tat-SOD on mouse skin was assayed with an IHC study. Finally, immunohistograms were taken with an Olympus DP-70 camera on a Nikon ECLIPSE E800 microscope (at an original magnification of × 200). **a** His-tag staining. **b** COX-2 protein staining. **c** Nitrotyrosine protein staining. Arrows indicate positive His-tag, COX-2, and nitrotyrosine staining which produced a brown-colored product

For the in vivo study, we examined the transduction and therapeutic evaluation of Tat-SOD in a murine skin model. The denatured Tat-SOD solution (1 or 5 μM) containing 0.05 mM CuCl_2_ and ZnCl_2_ was applied by uniformly dripping it onto the surface of exfoliated murine skin for 2 h and then examining the skin using IHC staining. The penetration signals of Tat-SOD were significantly detected in the epidermis and dermis probed with a Tat-specific antibody (Fig. 2a). Subsequently, we evaluated whether transduced Tat-SOD played a role as an antioxidant in vivo. Tat-SOD solution was topically administered to the shaven backs of mice with celecoxib (a COX-2 inhibitor) or PTIO (an SNP antagonist) for 30 min; then, animals were treated with SNP (an NO donor) for 4 h to test the protective effect. The effectiveness of Tat-SOD in inhibiting SNP-induced COX-2 and protein nitration was later measured using an IHC analysis. Results (Fig. 2b, c) showed that treatment with SNP (20 μmol) significantly increased levels of COX-2 and nitrotyrosine-containing proteins (brown pigment) in the epidermis. The pigment tone was significantly and dose-dependently attenuated by Tat-SOD and celecoxib, indicating that Tat-SOD effectively reduced SNP-induced COX-2 production as well as did celecoxib in mouse skin. Comparatively, the potency of 50 μg Tat-SOD was almost equivalent to that of 10 mg celecoxib. Further, Tat-SOD also successfully suppressed SNP-induced nitrotyrosine production in mouse skin via attenuating the production of ONOO^-^ by scavenging superoxide anions, demonstrating a detoxifying effect compared to PTIO.

**Fig. 3:**
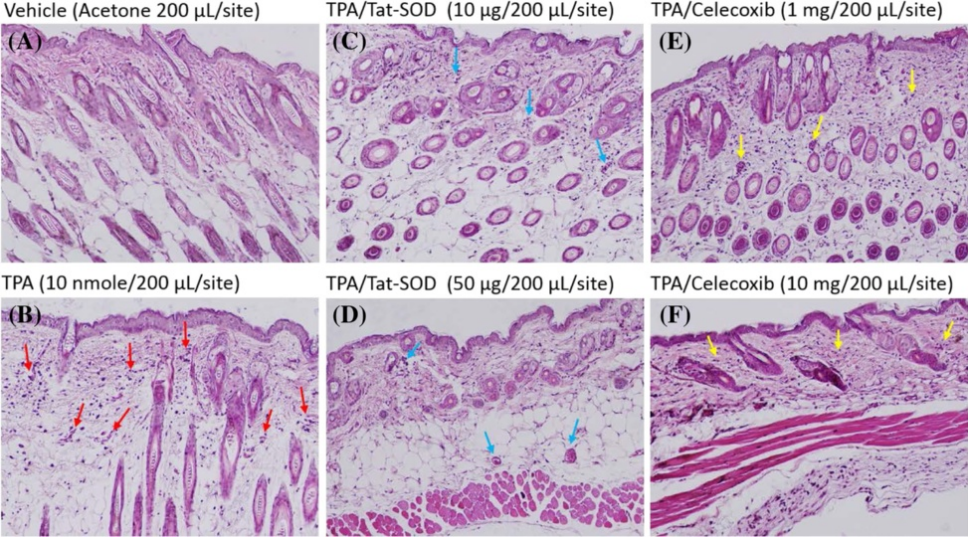
Inhibition of the TPA-induced mouse cutaneous inflammation by Tat-SOD. H&E-stained histological sections of a mouse skin sample obtained at indicated doses of Tat-SOD and celecoxib after a TPA (10 nmol/200 μL/site)-induced inflammatory response in mouse skin. Acetone-treated mice were used as the control. **a** Vehicle, (**b**) TPA, (**c-d**) TPA/Tat-SOD, and (**e, f**) TPA/celecoxib

Furthermore, we evaluated whether the Tat-SOD solution still played a role as an anti-inflammatory agent. Animals were challenged with TPA, which led to the excessive generation of ROS. Inflammation was induced by triple topical applications of TPA (20 μmole) on exfoliated murine back skin for 4 h; and later, Tat-SOD solution (1 or 5 μM) was topically administrated for 2 h. Histological staining with H&E (Fig. 3) showed that TPA induced the accumulation of inflammatory cells, vascular congestion, and neutrophil accumulation in the perivascular as a primary inflammatory phenomenon (Fig. 3b; red arrows). Tat-SOD administration obviously and dose-dependently reduced the accumulation of neutrophils (Fig. 3c, d; blue arrows). Celecoxib as a positive control only showed a minor primary inflammatory characteristic (Fig. 3e, f; yellow arrows). Results demonstrated that Tat-SOD solution was effective in attenuating TPA-induced mouse cutaneous inflammation, suggesting that Tat-SOD has high therapeutic benefits in blocking skin inflammation.

### Characterization of exogenous Tat-SOD function in vitro

**Fig. 4:**
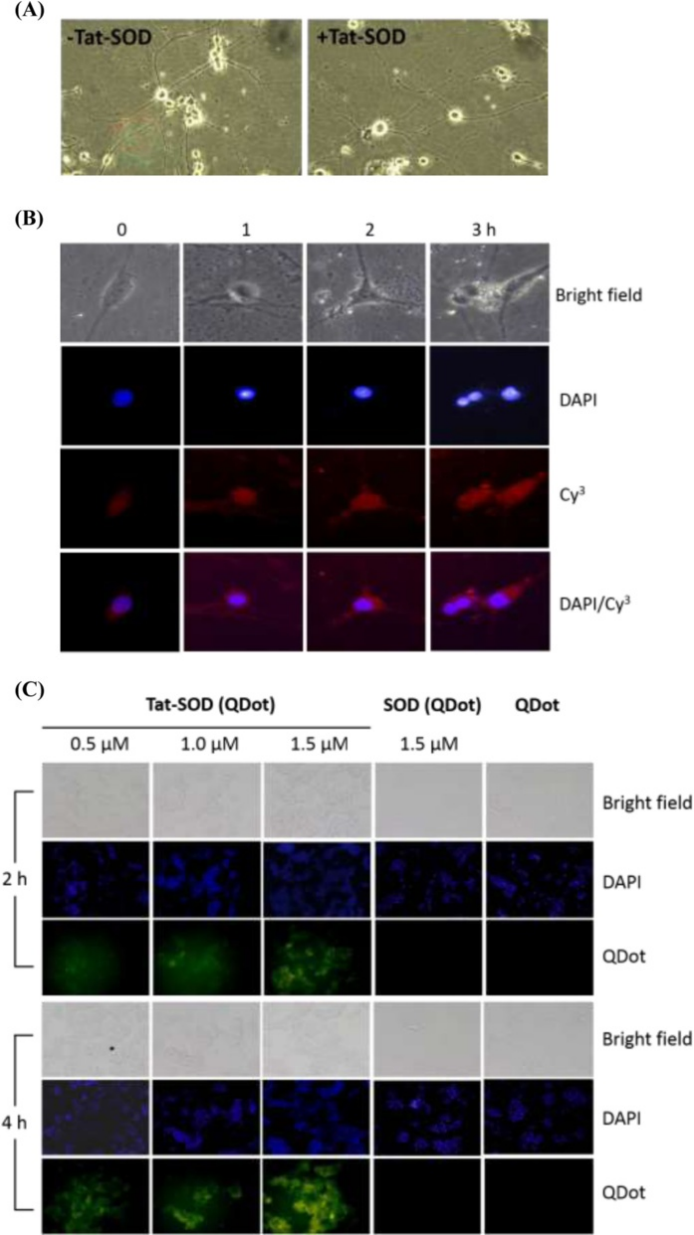
Transduction efficiency of denatured Tat-SOD into differentiated PC12 cells. **a** Differentiated PC12 cells plated in a six-well plate were treated with 1.5 μM of denatured TatSOD in the culture medium for 2 h. **b** Differentiated PC12 cells were incubated for various time intervals with 1.5 μM of denatured Tat-SOD. Then, the transduced fusion proteins in the cells were analyzed by immunofluorescence microscopy. DAPI, nuclear staining; Cy3, SOD1 staining. **c** PC12 cells were incubated with quantum dots (QDot 560 nm)-conjugated Tat-SOD molecules for 2 or 4 h with different doses of the conjugate (as indicated). Cells were later stained with DAPI. Results were observed using fluorescence microscopy with an FITC filter

The skin contains the peripheral endings of many of the sensory nerves. The injuries may affect to the neuronal cells caused by the subsequent inflammatory response. The penetration signals of Tat-SOD were also significantly detected in the epidermis and dermis in Fig. 2a. Here we chose PC12 cell, embryological origin of neuroblastic cells which differentiate into neuron-like cells and share properties similar to neurons, as a model to study if the denatured Tat-SOD solution was useful for cell protection in vitro. We induced the differentiation of PC12 cells with NGF (50 ng/mL) and then added the denatured Tat-SOD solution (1.5 μM) to the culture medium for 1, 2, and 3 h. Results (Fig. 4a) demonstrated that Tat-SOD-transduced PC12 cells grew well, which indicates that Tat-SOD did not cause cell death or morphologic changes. A penetration analysis assessed by immunofluorescence staining revealed that denatured Tat-SOD was successfully transduced into PC12 cells in a time-dependent manner (Fig. 4b). Instead of using indirect SOD antibody staining, photostable fluorescent quantum dots (QDots, 560 nm) were used to directly observe penetration into living cells. We conjugated Tat-SOD with thioglycolated QDots through a metal/His-tag interaction; then, QDot-Tat-SOD was introduced to the culture medium for 2 and 4 h. As shown in Fig. 4c, transduction only occurred after Tat-SOD treatment in a dose-dependent manner. With QDots only or QDots conjugated with SOD, cells showed no fluorescence uptake. Both results illustrated that the Tat-SOD protein was quickly transduced into cells within 2 h with time-dependent efficiency.

**Fig. 5:**
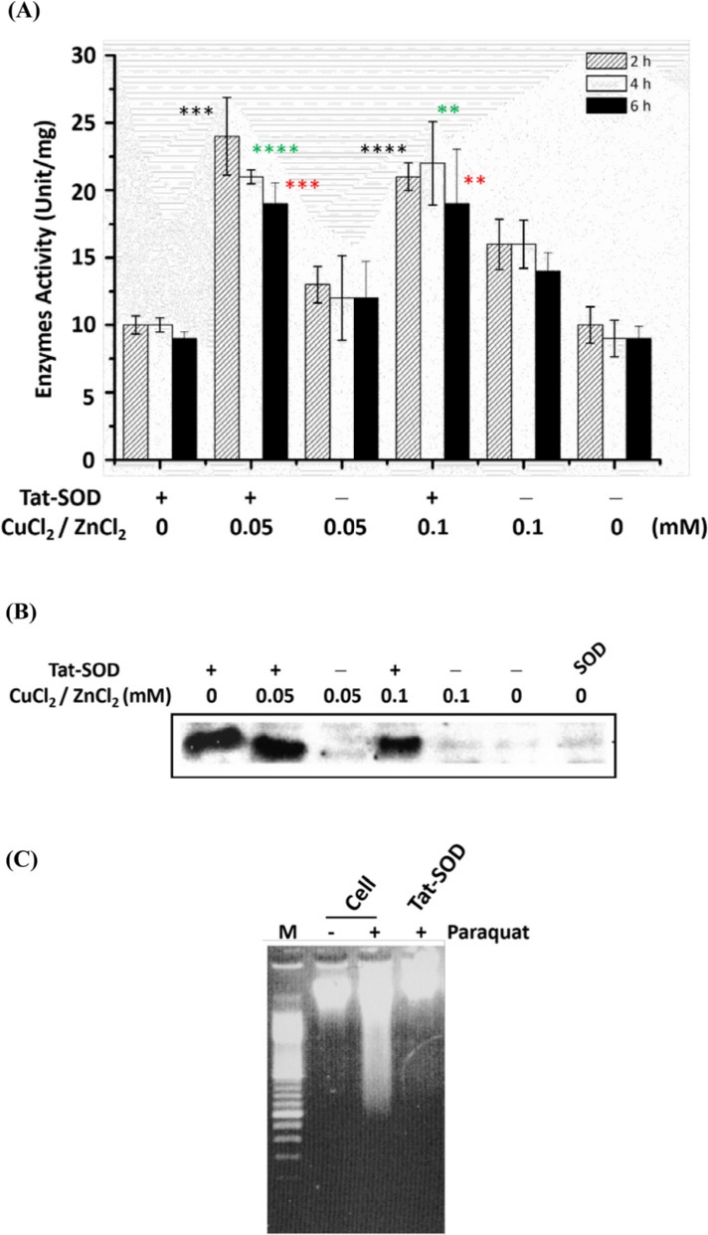
Denatured Tat-SOD transduced into PC12 cells retained its activity via protein refolding. PC12 cells were treated with or without Tat-SOD (1.5 μM) and incubated in 0, 0.05 or 0.1 mM of CuCl_2_ and ZnCl_2_, for 2, 4 or 6 h, then cells were lysed. **a** Enzymatic assay of SOD. SOD-specific activity was analyzed by the pyrogallol method (as described in “Materials and Methods”). **b** Cellular transduction of SOD by Tat-SOD. Cellular SOD protein expression levels were analyzed by Western blotting. SOD only (without Tat-tagged) was used for the control. **c** Tat-SOD protected PC 12 cells from superoxide anion attack generated by 70 mM paraquat. After treatment, DNA samples were isolated from PC12 cells and then separated on a 1 % agarose gel. Lane 1, DNA marker; lane 2, cell only; lane 3, paraquat treatment; lane 4, Tat-SOD (1.5 μM) transduced with paraquat. Each experiment was performed in triplicate. All columns displayed as mean ± SEM Significance was calculated compared to controls and *p* values were calculated by applying Student’s *t*-test. For the additions of Tat-SOD, statistically very significant differences from added 0.05 or 0.1 mM metals (ZnCl_2_/CuCl_2_) compared to 0 mM metal control within 2 h (***p < 0.001; ****p < 0.0001, respectively), 4 h (**** *p* < 0.0001; **p < 0.01, respectively) or 6 h (***p < 0.001; **p < 0.01, respectively); but, statistically no significant difference from 0.5 to 1.0 mM metals added (*p* > 0.05) within 2, 4 or 6 h. For the no additions of Tat-SOD, statistically no significant differences between added 0.05 or 0.1 mM (ZnCl_2_/CuCl_2_) metals compared to 0 mM metal control within 2 h (*p < 0.05; **p < 0.01, respectively), 4 h (ns, *p* > 0.05; **p < 0.01, respectively) or 6 h (***p < 0.001; **p < 0.01, respectively). For the additions added of Tat-SOD within 2, 4 or 6 h with 0 mM metals (ZnCl_2_/CuCl_2_), statistically significant differences from 2 h compared to 4 or 6 h with no significant differences (*p* > 0.05), 4 h compared to 6 h with *p < 0.05; with 0.05 mM metals (ZnCl_2_/CuCl_2_), statistically significant differences from 4 h compared to 2 or 6 h with no significant differences (*p* > 0.05), 2 h compared to 6 h with *p < 0.05; but, added 0.1 mM metals (ZnCl_2_/CuCl_2_) within 2, 4 or 6 h statistically no significant differences (*p* > 0.05)

Restoration of the enzymatic activity of cells is critical after proteins are transduced. Herein, we showed that the transduced Tat-SOD protein regained its activity in PC12 cells. PC12 cells were cultured with 1.5 μM of Tat-SOD and 0, 0.05 or 0.1 mM of CuCl_2_ and ZnCl_2_ for 3 h. Cell lysates were analyzed for SOD enzyme activity, and Western blotting was used to determine SOD protein levels. Results (Fig. 5a) showed that Tat-SOD co-cultured with 0.05 or 0.1 mM CuCl_2_ and ZnCl_2_ had higher SOD activities compared to Tat-SOD alone. The Western blotting analysis (Fig. 5b) showed that the addition of metal ions did not interfere with the transduction efficiency. Meanwhile, native SOD could not enter cells without the Tat-transducing peptide. Results implied that the molecular refolding to gain activity occurred inside cells by cellular machinery and involved metal ions.

In addition to evaluating the antioxidant ability, PC12 cells transduced with the Tat-SOD protein were then treated with paraquat (70 mM), an intracellular superoxide anion generator. Results (Fig. 5c) showed that the addition of paraquat induced abundant DNA fragmentation via cell apoptosis, and the Tat-SOD protein prevented this fragmentation, demonstrating that Tat-SOD effectively protected PC12 cells against attack by superoxide anions. Even though NGF protects neuronal cells from superoxide anion damage [34], ROS-mediated apoptotic damage has also found in the differentiated cells and could be diminished by the Tat-SOD protein.

**Fig. 6:**
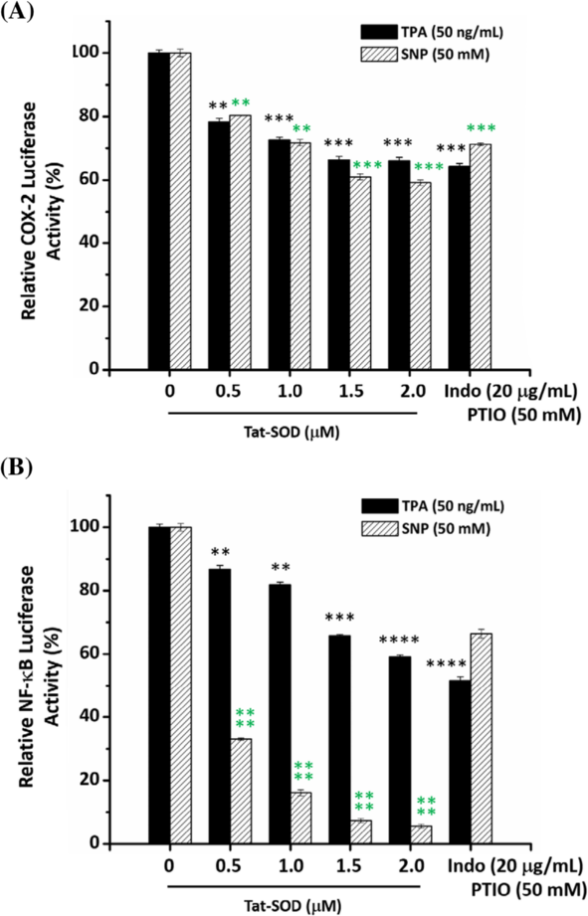
Effect of Tat-SOD on TPA- or SNP-induced promoter activity of cyclooxygenase-2 (COX-2) or nuclear factor–κB (NF-κB) in PC12 cells. PC12 cells were transfected with a luciferase reporter plasmid of (**a**) COX-2 or (**b**) NF-κB for 24 h, then incubated with Tat-SOD, indomethacin (Indo), or PTIO for 2 h. After being stimulated for 4 h with TPA (50 ng/mL) or SNP (50 mM), luciferase activity was measured, and COX-2 and NF-κB activities were expressed relative to cells treated with TPA or SNP alone. Each experiment was performed in triplicate. Data are represented as mean ± SEM. Significance was calculated compared to controls and *p* values were calculated by applying Student’s *t*-test. For the relative COX-2 luciferase activity, statistically significant difference at exposed to TPA (50 ng/mL) + Tat-SOD (0.5 μM) compared to TPA control (50 ng/mL) (**p < 0.01), very significant differences at exposed to TPA (50 ng/mL) + Tat-SOD (1.0, 1.5, 2.0 μM) or Indo (20 μg/mL) compared to TPA control (50 ng/mL) (***p < 0.001); also, statistically significant differences at exposed to SNP (50 mM) + Tat-SOD (0.5 or 1.0 μM) compared to SNP control (50 mM) (**p < 0.01), very significant differences at exposed to SNP (50 mM) + Tat-SOD (1.5 or 2.0 μM) or PTIO (50 mM) compared to SNP control (50 mM) (***p < 0.001). For the relative NF-κB Luciferase activity, statistically significant difference at exposed to TPA (50 ng/mL) + Tat-SOD (0.5 or 1.0 μM) compared to TPA control (50 ng/mL) (**p < 0.01), very significant differences at exposed to TPA (50 ng/mL) + Tat-SOD (1.5 μM) compared to TPA control (50 ng/mL) (***p < 0.001), and very significant differences at exposed to TPA (50 ng/mL) + Tat-SOD (2.0 μM) or Indo (20 μg/mL) compared to TPA control (50 ng/mL) (****p < 0.0001); also, statistically significant differences at exposed to SNP (50 mM) + Tat-SOD (0.5, 1.0, 1.5, 2.0 μM) compared to SNP control (50 mM) (****p < 0.0001), very significant differences at exposed to SNP (50 mM) + PTIO (50 mM) compared to SNP control (50 mM) (***p < 0.001)

Furthermore, results of Tat-SOD inhibiting SNP-induced COX-2 expression in vivo implied the involvement of a cellular signal transduction pathway. Previous studies revealed that abnormal expressions of NF-κB and COX-2 play a crucial role in ROS-related diseases [6, 8, 22, 35]. We further examined whether the inhibitory mechanism of Tat-SOD was related to NF-κB and COX-2 at the transcriptional level. We transfected reporter constructs containing COX-2 (Fig. 6a) and NF-κB (Fig. 6b) promoters into differentiated PC12 cells and measured the promoter activity with a luciferase reporter assay. As observed, treatment of transfected cells with TPA or SNP significantly increased NF-κB and COX-2 luciferase activities. When pretreated with Tat-SOD, TPA- or SNP-stimulated luciferase activity was dose-dependently inhibited. The addition of 0.5–2 μM Tat-SOD suppressed COX-2 promoter activity by 60–80 % compared to TPA or SNP alone. Indo and PTIO as positive controls suppressed COX-2 promoter activities by 64 and 71 %, respectively. The same doses of Tat-SOD also inhibited NF-κB promoter activities by 59–87 % after activation with 50 ng/mL TPA and by 5–33 % after activation with 50 mM SNP. Indo and PTIO as references suppressed NF-κB promoter activities by 52 and 66 %, respectively. Together, these results suggest that Tat-SOD suppresses COX-2 expression and NF-κB activity via regulating promoter activities.

## Discussion

Oxidative stress is defined as an impaired balance between ROS production and antioxidant defenses and is involved in many diseases [36]. Removing ROS can be an effective precautionary measure against pathogenesis by exogenous SOD. SOD1, an oligomeric protein with Cu^2+^ and Zn^2+^, has a two-domain structure: one domain contains α-helices and the second is composed of both α-helices and β-sheets [37]. Metal-binding sites are located between two domains with side chains of histidine and aspartate. In this paper, we demonstrated that the addition of heavy metals caused the loss of zinc, resulting in conformational changes of α-helices and β-sheets. It also implied that if the formulation was combined with protective chelating agents in the ointment, such as Na_2_EDTA or diethyldithiocarbamate, this would reduce the binding of Cu^2+^ and Zn^2+^ in SOD molecules [38]. Improper vehicles such as large quantities of water or vaselinum album also caused rapid instability of the native SOD. Therefore, the strategy of using denatured protein makes preparation and formulation much easier and simpler by avoiding the risk of deactivating the native form and loss of ligand metals. The intracellular delivery of macromolecules still remains problematic because of the bioavailability restriction imposed by their intrinsic poor stability, membrane permeability, and endosomal trapping/degradation. Some experiments demonstrated the efficacy of oral SOD supplements or administration by subcutaneous, intravenous, intraperitoneal, or intramuscular injections. However, all these routes have limitations with transducing a protein into cells or the circulation, and almost all uses of native SOD could be still unnecessarily true [39]. The design of Tat-SOD contains a Tat domain (RKKRRQRRR) at the NH_2_-terminus [40]. Using cell-penetrating peptides to carry the peptides into cells was demonstrated to bypass the barrier in animal and culture models [41]. Over 50 proteins of different sizes were reported in human or murine cell types in vitro [42–45]. We herein illustrated that the Tat-SOD penetrated into murine cells which was independent of receptors and transporters [46], and via an endosome escape mechanism [29].

In the present work, we assessed the effects of TPA on inducing inflammation in mouse skin. Our results of H&E histostaining agreed with TPA-induced vascular congestion and neutrophil accumulation in the perivascular region observed as a primary inflammatory reaction. Tat-SOD effectively attenuated cutaneous inflammation, suggesting that Tat-SOD affords a high therapeutic benefit in skin disorders, such as neutrophil dermatose and cutaneous inflammation [47]. Similar to TPA, SNP-generated oxidative stress induced expressions of iNOS and COX-2 and stimulated NF-κB migration associated with the degradation of IκBα [48]. Our results showed that topical administration of Tat-SOD inhibited SNP-induced COX-2 expression as well as did celecoxib. Celecoxib, a selective COX-2 inhibitor, is widely used to treat rheumatoid arthritis, psoriatic arthritis, adenomatous polyposis, and osteoarthritis [49, 50]. Compared to celecoxib, Tat-SOD exhibited a similar potency in dose-dependently inhibiting COX-2 expression through a different mechanism. For SNP-induced upregulation of iNOS, the presence of NO produced by reacting with superoxide anions leads to the formation of peroxynitrites, which immediately interact with tyrosine residues of proteins to form nitrotyrosines at a rate of 6.7×10^9^ M^-1^ s. PITO serves for a stable NO scavenger without affecting NOS activity and significantly inhibits NO biological actions. In our study, Tat-SOD scavenged superoxide anions rather than NO, to attenuate the formation of nitrotyrosines. This suggests that applying Tat-SOD without blocking NO’s biological activities can prevent peroxynitrite-related diseases, such as sunburn erythema, contact hypersensitivity, and poly (ADP-ribose) polymerase-mediated diseases [51].

## Conclusions

In conclusion, we developed an effective therapeutic application using the denatured Tat-SOD solution, a newly modified form of SOD, which could overcome the instability of native SOD formulations and side effects of anti-inflammatory agents. Tat-SOD was efficiently delivered in vivo with restoration of biological activity in mammalian tissues. Topical administration of Tat-SOD solution exhibited a dual effect of suppressing the formation of peroxynitrite and the expression of COX-2 in murine skin. This success lays the groundwork for eventual transduction of therapeutic proteins into patients with ROS-mediated diseases.
